# Integrating Radiosensitivity Gene Signature Improves Glioma Outcome and Radiotherapy Response Prediction

**DOI:** 10.3390/medicina58101327

**Published:** 2022-09-22

**Authors:** Shan Wu, Jing Xu, Guang Li, Xi Jin

**Affiliations:** 1 Department of Radiation Oncology, The First Hospital of China Medical University, Shenyang 110001, China; 2 Key Laboratory of Networked Control Systems, Chinese Academy of Sciences, Shenyang 110016, China; 3 Shenyang Institute of Automation, Chinese Academy of Sciences, Shenyang 110016, China; 4 Institutes for Robotics and Intelligent Manufacturing, Chinese Academy of Sciences, Shenyang 110169, China

**Keywords:** glioma, radiosensitivity, gene signatures

## Abstract

Response to radiotherapy (RT) in gliomas varies widely between patients. It is necessary to identify glioma-associated radiosensitivity gene signatures for clinically stratifying patients who will benefit from adjuvant radiotherapy after glioma surgery. **Methods:** Chinese Glioma Genome Atlas (CGGA) and the Cancer Genome Atlas (TCGA) glioma patient datasets were used to validate the predictive potential of two published biomarkers, the radiosensitivity index (RSI) and 31-gene signature (31-GS). To adjust these markers for the characteristics of glioma, we integrated four new glioma-associated radiosensitivity predictive indexes based on RSI and 31-GS by the Cox analysis and Least Absolute Shrinkage and Selection Operator (LASSO) regression analysis. A receiver operating characteristic (ROC) curve, integrated discrimination improvement (IDI), and net reclassification improvement (NRI) were used to compare the radiosensitivity predictive ability of these six gene signatures. Subgroup analysis was used to evaluate the discriminative capacity of those gene signatures in identifying radiosensitive patients, and a nomogram was built to improve the histological grading system. Gene Ontology (GO) analysis and Gene Set Enrichment Analysis (GSEA) were used to explore related biological processes. **Results:** We validated and compared the predictive potential of two published predictive indexes. The AUC area of 31-GS was higher than that of RSI. Based on the RSI and 31-GS, we integrated four new glioma-associated radiosensitivity predictive indexes—PI10, PI12, PI31 and PI41. Among them, a 12-gene radiosensitivity predictive index (PI12) showed the most promising predictive performance and discriminative capacity. Examination of a nomogram created from clinical features and PI12 revealed that its predictive capacity was superior to the traditional WHO classification system. (C-index: 0.842 vs. 0.787, *p* ≤ 2.2 × 10^−16^) The GO analysis and GSEA showed that tumors with a high PI12 score correlated with various aspects of the malignancy of glioma. **Conclusions:** The glioma-associated radiosensitivity gene signature PI12 is a promising radiosensitivity predictive biomarker for guiding effective personalized radiotherapy for gliomas.

## 1. Introduction

Glioma, one of the most common primary brain malignancies in adults, is characterized by high recurrence rates and fatality [1]. Surgical resection and chemoradiotherapy are the main glioma treatments. However, glioma is heterogeneous and complex, with widely varied clinical outcomes. Despite the best available treatments, glioma survival ranges from weeks/months to years [2]. Based on an improved understanding of its molecular features, glioma was reclassified based on histological and molecular diagnostic criteria [3]. The rapidly evolving understanding of the molecular subtypes of glioma has numerous clinical implications. However, most studies have focused on sensitivity to chemotherapy or novel targeted therapies. Thus, a better understanding of the hallmarks of radiotherapy, which is the most common and cost-effective adjuvant therapy, is needed for effective personalized radiotherapy.

Several attempts have been made to identify a robust gene signature to predict the radiosensitivity. The radiosensitivity index (RSI) and 31-gene signature (31-GS) are burgeoning approaches for pan-cancer analysis. The RSI is a 10-gene model based on the radiation survival at 2Gy (SF2) in 48 human cancer cell lines [4]. The prediction model is a linear regression algorithm that is validated in esophageal cancer, head and neck cancer, rectal cancer, breast cancer, and endometrial cancer [5,6,7]. The 31-GS is developed by analyzing the NCI-60 cancer cell panel for genes whose expression correlates with SF2 [8]. Its association with radiosensitivity has been validated in various malignancies [9,10,11]. However, it is still unclear which one is more suitable for glioma. Notably, there are no overlapping genes between the two radiosensitivity predictive indexes. Exactly which genes play an important predictive role in glioma also remains unknown.

Here, we compared the ability of the two gene signatures to predict glioma radiosensitivity using Chinese Glioma Genome Atlas (CGGA) and the Cancer Genome Atlas (TCGA) glioma patient datasets. Four new integrating radiosensitivity gene signatures were established based on the two signatures and adapted to glioma. Sensitivity and specificity were used to assess the reliability and accuracy of these radiosensitivity biomarkers. A nomogram involving optimal radiosensitivity gene signature was built for glioma prognosis. The concordance index (C-index), calibration plots, and decision curve analyses (DCA) showed that this signature is superior to the conventional WHO staging system alone.

## 2. Methods

### 2.1. Public Data Acquisition

Datasets on 748 and 647 glioma cases were obtained from CGGA (http://www.cgga.org.cn/, accessed on 9 June 2019) and TCGA (http://xenabrowser.net/, accessed on 8 March 2020), respectively. Transcriptome sequencing data were generated on an Illumina Hiseq platform. Patient demographic and clinicopathological features are shown in Table 1.

### 2.2. Validation of the Two Existing Radiosensitivity Signatures

The CGGA and TCGA datasets were divided into two groups based on the expression profile of the 31-gene signature (31-GS) using the k-means method. Clusters with poor progress were defined as radioresistant (RR) groups (CGGA-RR and TCGA-RR). The remaining two groups were designated as radiosensitive (RS) groups (CGGA-RS group and TCGA-RS group).

RSI was calculated as follows: RSI = −0.0090008 × AR expression + 0.0128283 × JUN expression + 0.0254552 × STAT1 expression − 0.0017589 × PRKCB expression − 0.0038171 × RELA expression + 0.1070213 × ABL1 expression − 0.0002509 × SUMO1 expression − 0.0092431 × PAK2 expression − 0.0204469 × HDAC1 expression − 0.0441683 × IRF1 expression. According to Eschrich et al. [4], the lower quartile of RSI was pre-defined as the cut-off point for dividing patients into the RS and RR groups.

### 2.3. Generating Improved Radiosensitive Models

Both RSI and 31-GS are based on various cell lines and are not glioma-specific. To identify precise glioma radiosensitivity signatures, we used the CGGA dataset as a training cohort and the TCGA cohort as a validation cohort. Cox and LASSO analyses were used to predict glioma overall survival with radiotherapy (*n* = 625). The models were reformulated as follows:

(1) $$PIn=\sum_{k=1}^{n}\left(\mathrm{Expk}*\mathrm{Coek}\right),$$

where *n* is the number of selected genes, Expk is the expression value for each gene, and Coek is the coefficient of regression models. This analysis identified four improved radiosensitivity predictive indexes (PIx)—PI10 (involving genes in the RSI model), PI31 (involving genes in the 31-GS model), PI41 (involving genes from the two models), and PI12 (involving the 12 genes identified via LASSO regression analysis).

### 2.4. Validation and Comparison of the Prognostic Indexes

For the internal and external validation of our PIs, we used time-dependent receiver operating characteristic (ROC) curve analysis to determine corresponding cut-off points and divide patients into high- and low-level groups. The areas under ROC curves (AUCs) were compared to determine the PIs’ prognostic potential. Net reclassification improvement (NRI) and integrated discrimination improvement (IDI) were used to estimate the improvement of the model’s predictive power upon risk factors addition. AUC, NRI, and IDI were then used to evaluate the discriminative ability of the selected PIs when predicting glioma outcomes. The IDI and NRI values of the selected six predictors were calculated using the R “SurvivalIDI package”. RSI was used as a reference.

### 2.5. Comparing the Discrimination Capability of the Selected Radiosensitivity Markers

To determine patients who can benefit from radiotherapy (RT), we performed Cox analysis to assess the discriminability of each radiosensitivity predictor in the RT and non-RT groups. Cox analysis was used to determine which group should receive radiotherapy. Given that glioma is characterized by diverse histological types, Cox analysis was conducted on patients with various histology types. Both CGGA and TCGA glioma cases were tested by Cox analysis, and the subgroup analysis results were shown in forest plots. Discriminability and clinical application potential were examined based on sensitivity and specificity. Predictors with optimal specificity only exhibited prognostic capacity in the RT group, while those with optimal sensitivity were capable of distinguishing patients who may benefit from RT from those who can be spared RT.

### 2.6. Nomogram Construction and Analysis of Its Performance against the WHO Staging System

After comparing the predictive capacity of the selected radiosensitivity predictors, the optimal ones were included in a nomogram. Next, Cox regression univariate and multivariate analyses were performed using the CGGA dataset as a training cohort and the TCGA dataset as a validation cohort. A nomogram was built based on multivariate analysis results of the training cohort and validated on the validation cohort using a concordance index (C-index), calibration plot, and decision curve analysis (DCA).

### 2.7. Exploration of Biological Function

**Figure 1:**
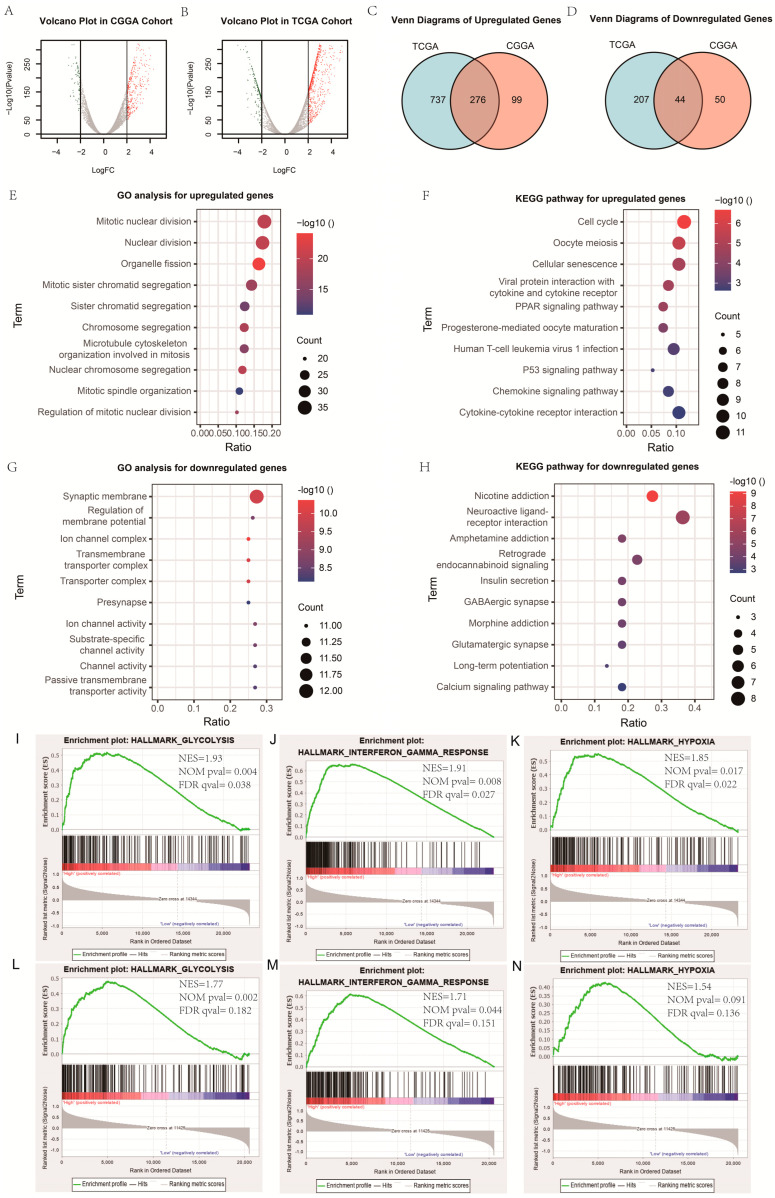
Exploration of biological function. (**A**,**B**) Volcano plot of gene expression profile data in high-PI12 samples and low-PI12 ones in CGGA and TCGA cohort; (**C**,**D**) Venn diagram of up-regulated and down-regulated differentially expressed genes (DEGs); (**E**,**G**) Bubble plots of the top ten GO terms of biological processes based on the up-regulated and down-regulated genes; (**F**,**H**) Bubble plots of the top ten KEGG pathways based on the up-regulated and down-regulated genes; (**I**–**K**) Gene set enrichment analysis (GSEA) showed the biological functions and pathways that were significantly enriched in patients with high-PI12 score in CGGA cohort; (**L**–**N**) The biological functions and pathways that were significantly enriched in TCGA cohort.

To explore the underlying biological mechanisms related to differences in radiosensitivity, differential expressed genes (DEGs) between the high-risk and low-risk groups were separately identified using the “edgeR” R package. When there was a false discovery rate (FDR) q < 0.01 and fold change (FC) > 2.0, significant genes were defined (Figure 1A,B). Similarities of significant genes in both the CGGA and TCGA cohorts were classified as significant DEGs (Figure 1C,D). The “clusterProfiler” R package was used to carry out Gene Ontology (GO) analysis and Kyoto Encyclopedia of Genes and Genomes pathway enrichment (KEGG) analysis [12]. Gene Set Enrichment Analysis (GSEA) was used to uncover the mechanism of radioresistance (version 4.0.1).

To validate the results of these bioinformatics analyses, we tested the correlation between the PI12 and several published biological-related scores, such hypoxia score, glycolysis-related score and interferon-γ-related score. Hypoxia scores were calculated based on the mRNA-abundance-based signature developed previously by Buffa et al. [13]. Patients with the top 50% of mRNA abundance values for each gene in a signature were given a score of +1, otherwise they were given a score of −1. The glycolysis-related score was calculated based on the specific formula: Risk score = (0.1730 × SOCS3 expression) + (0.2370 × ISG20 expression) + (−0.4303 × IFIT5 expression) + (−0.2914 × NLRC5 expression) + (−0.2829 × IRF9 expression) [14]. The interferon-γ related score was calculated based on the specific formula: Risk score = (0.19 × FOXD2-AS1 expression) + (−0.27 × AC062021.1 expression) + (−0.16 × AF131216.5 expression) + (−0.05 × LINC00844 expression) + (0.11 × CRNDE expression) + (0.35 × LINC00665 expression) [15].

### 2.8. Statistical Analyses

Statistical analyses were conducted on R Studio (version 1.1453). Survival curves were generated using Kaplan–Meier analysis and analyzed using the log-rank test. Cox regression analysis was used for univariate and multivariate analyses. Heatmap analysis was performed using the *pheatmap* package. Correlation heatmap analysis was performed using the *ggcor* package. The LASSO model was built using the *glmnet* package. The *SurvivalROC* package was used for ROC curve analysis. The *SurvivalIDI package* was used for IDI and NRI analysis. The nomogram was built using the *rms* package. DCA was performed using the *stdca* package.

## 3. Results

### 3.1. Validation of the Predictive Capacity for RSI and 31-Gene Signatures

**Figure 2:**
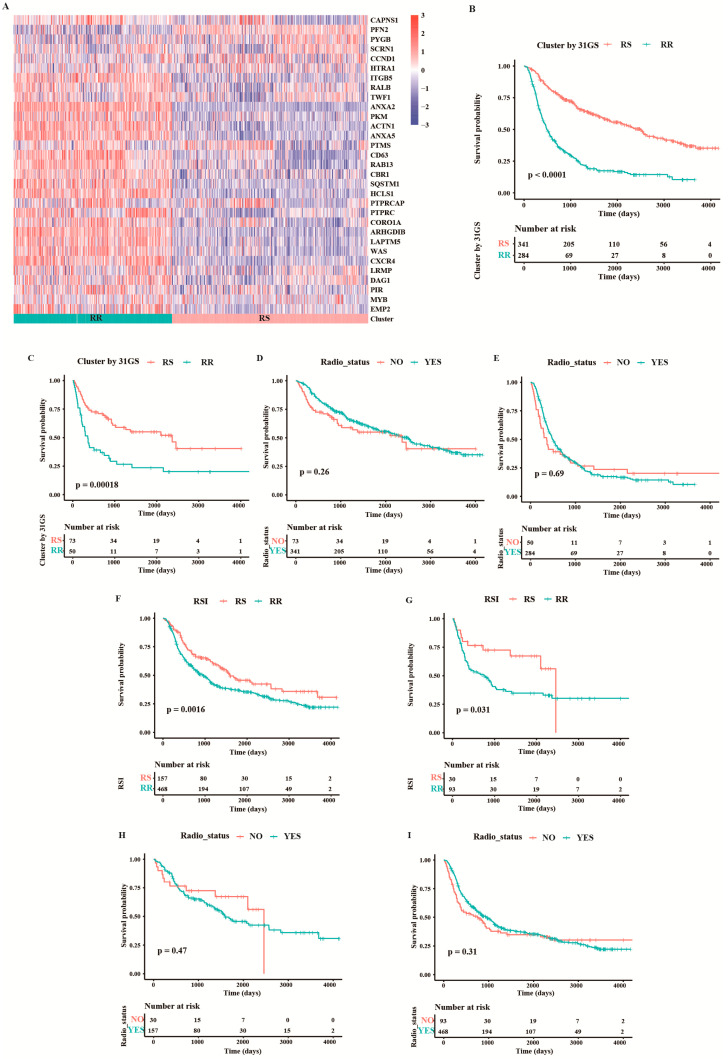
Validation of the two existing radiosensitivity signatures in CGGA cohort. (**A**) Heatmap illustrating the condition of 31-gene signature and radiosensitivity classification in all samples from the CGGA cohort. Survival comparison between RS group and RR group for patients treated with (**B**) and without RT (**C**) when classifying them based on 31-GS. Survival comparison between patients with and without RT in the RS group (**D**) and RR group (**E**) classified based on 31-GS. A comparison of overall survival between RS and RR group for patients treated with (**F**) and without RT (**G**) based on RSI. Comparison of overall survival between patients with and without RT in the RS group (**H**) and RR group (**I**) classified based on RSI.

First, we validated the predictive capacity of the 31-GS in the CGGA cohort and grouped the patients into the RR (*n* = 414) and RS (*n* = 334) groups using k-means clustering (Figure 2A). The group with improved survival was defined as the RS group. We then showed that the RS group exhibited better survival relative to the RR group whether or not they received RT (Figure 2B,C). No survival difference was observed between the RT and non-RT group irrespective of the group the patients belonged to (Figure 2D,E).

According to Eschrich et al. [4], patients on the lower RSI quartile are considered to be in the RS group, and the rest in the RR group. Patients in the RS group exhibited better survival than those in the RR group with or without RT (Figure 2F,G). Consistent with the 31-GS, no survival difference was observed between RT and non-RT group (Figure 2H,I), indicating that RSI did not determine which patients may benefit from radiotherapy or those who could be spared of RT.

### 3.2. Integrating Radiosensitive Genes Improves Radiosensitivity Prediction

**Figure 3:**
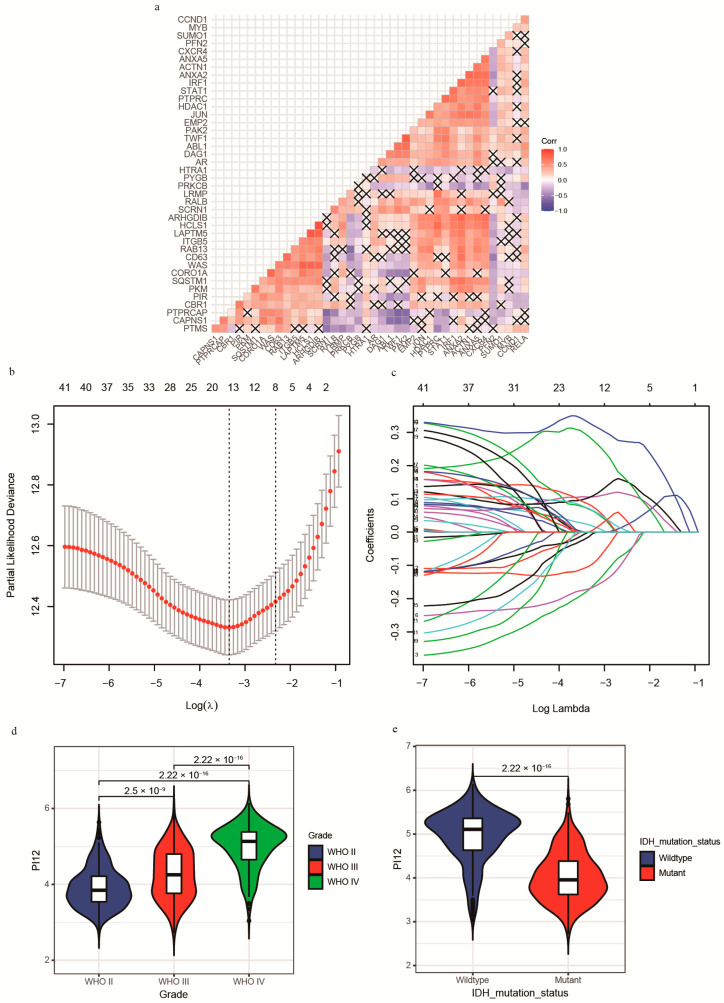
Construction of Lasso−cox model and PI12. (**a**) The correlation heatmap between radiosensitivity genes; (**b**) Partial likelihood deviance of different numbers of variables revealed by the LASSO regression analysis. The two vertical lines represent lambda.min and lambda.1se, respectively; (**c**) Log (Lambda) value of the 41 genes in LASSO regression analysis; (**d**) Distribution of the PI12 in glioma patients stratified by WHO grade; (**e**) Distribution of the PI12 in glioma patients stratified by IDH mutation status in the CGGA cohort.

Separately, the 31-GS and RSI could not robustly identify radiosensitive populations. Considering that both gene signatures were developed from various cell lines and are not glioma-specific, we rebuilt the PIs based on glioma patients with RT. First, the 41 genes were analyzed using univariate Cox regression. The results showed that not all genes were prognostic factors of glioma, shown in Table 1. Moreover, correlation analysis revealed a high correlation between some of the genes (Figure 3A), highlighting the difficulty of identifying significant genes using traditional statistical methods. The shrinking of the numbers of genes is necessary; thus, we used LASSO regression analysis to identify important predictive genes (Figure 3B). To obtain a more accurate prognostic model, we defined λ as the lambda.min (Figure 3C). As a result, 12 corresponding gene numbers were involved. These genes’ coefficients obtained from univariate and LASSO analysis are shown in Table 2. Based on the coefficients, we built PI12 and investigated the association between PI12 score and pathological characteristics. In the training cohort, PI12 score rose with the increase in WHO grades (Figure 3D). The IDH wildtype patients also showed higher PI12 scores than the IDH mutant ones (Figure 3E).

### 3.3. Validation and Comparison of the Prognostic Indexes

**Figure 4:**
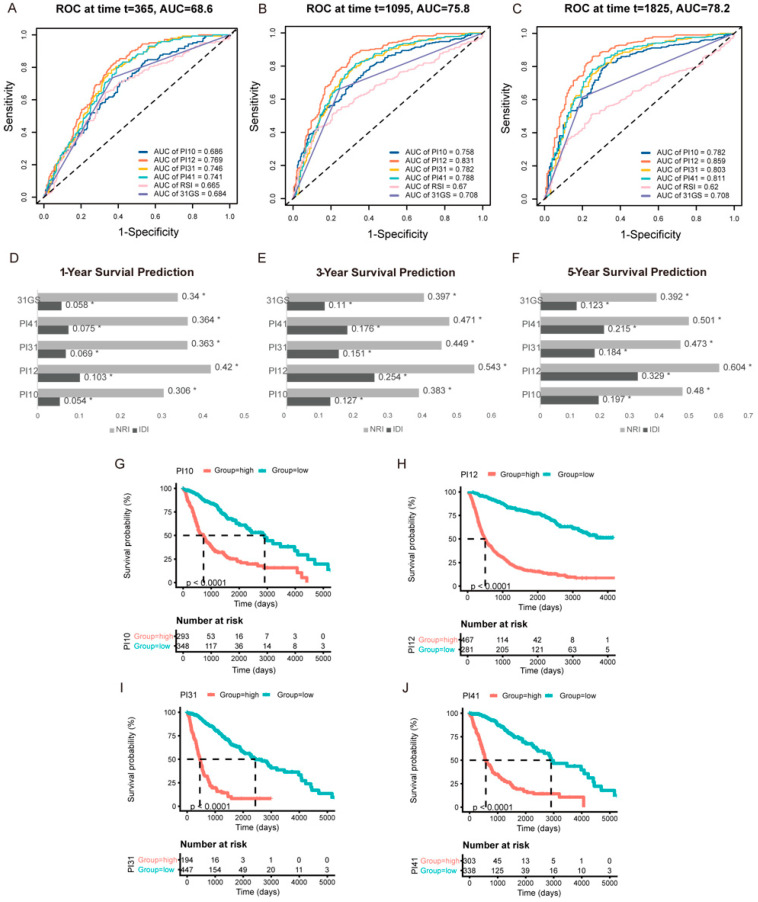
Internal validation of the selected markers in predicting glioma outcome in CGGA cohort. One−year (**A**), three−year (**B**), and five−year (**C**) time−dependent ROC of the selected markers. AUC areas were calculated and exhibited below the figures. IDI and NRI were calculated to compare the predictive accuracy of the 6 selected markers for 1-year (**D**), 3−year (**E**), 5−year (**F**) ROC. RSI was applied as the referent marker. * *p* < 0.05. (**G**–**J**) Prognostic significance of PI10, PI12, PI31, and PI41 score in CGGA cohort.

For internal validation, ROC curves were used to determine the cut-off thresholds. The AUCs were also compared to determine the prognostic potential of these PIx scores. The time-dependent ROC curves for 1-, 3-, and 5-year survival in the CGGA cohort are shown in Figure 4A–C. At all the time points, the PI12 score had the highest AUC, followed by PI31 and PI41. To compare the predictive accuracy of the selected six markers, we performed IDI and NRI analysis. As RSI’s AUC was the lowest, RSI was chosen as a referent marker. The other five predictors exhibited significant capacity to predict survival (*p* ≤ 0.05). Consistent with the ROC curve results, PI12 had the highest IDI and NRI value at all time points, followed by PI31 and PI41 (Figure 4D–F). K m curve analyses were performed to display the prognostic value of these prognostic indexes. The results showed that patients in the high-level prognostic index group tended to have shorter OS (Figure 4G–J).

**Figure 5:**
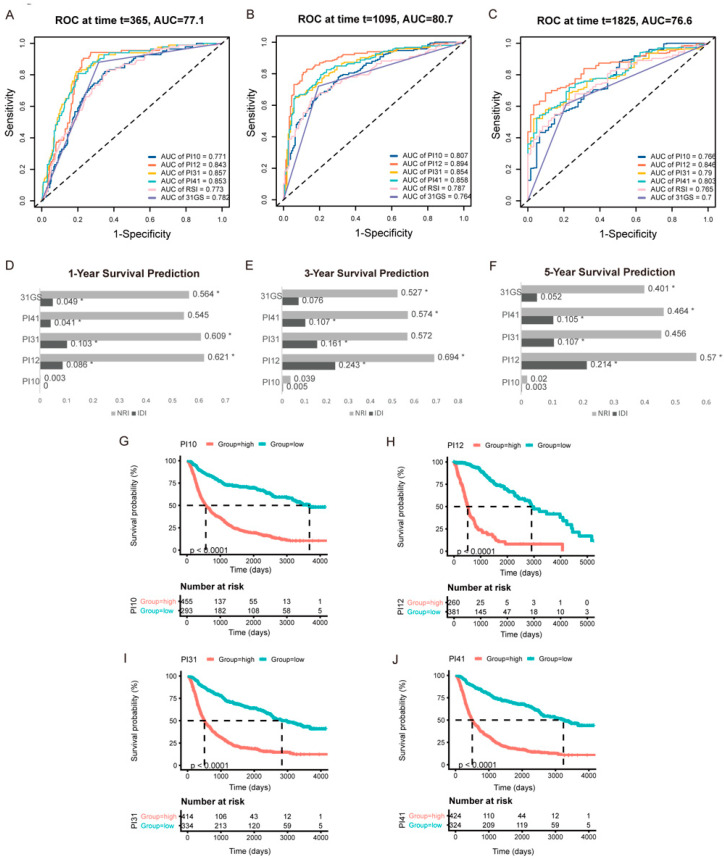
External validation of the selected markers in predicting glioma outcome in TCGA cohort. One−year (**A**), three−year (**B**), and five−year (**C**) time-dependent ROC of the selected markers. AUC areas were calculated and exhibited below the figures. IDI and NRI were calculated to compare the predictive accuracy of the 6 selected markers for 1−year (**D**), 3−year (**E**), 5−year (**F**) ROC. RSI was applied as the referent marker. * *p* < 0.05. (**G**–**J**) Prognostic significance of PI10, PI12, PI31, and PI41 score in TCGA cohort.

Similar results were obtained from external validation (Figure 5). Time-dependent ROC curves for 1−, 3−, and 5−year survival in the TCGA cohort are shown in Figure 5A–C. PI12 had the highest AUC for 3− and 5−year survival, followed by PI31 for 1−year survival. PI12 had the highest NRI at all time points. IDI results had the same trend as AUC values. PI12’s IDI value was highest at 3- and 5-year time points, followed by those of the PI31 model (Figure 5D–F). K m curve analyses also showed that high−level groups evaluated by all the prognostic indexes appeared to have a worse prognosis than those with the low-level counterparts (Figure 5G–J).

### 3.4. Comparing the Discrimination Capability of the Selected Radiosensitivity Predictors

**Figure 6:**
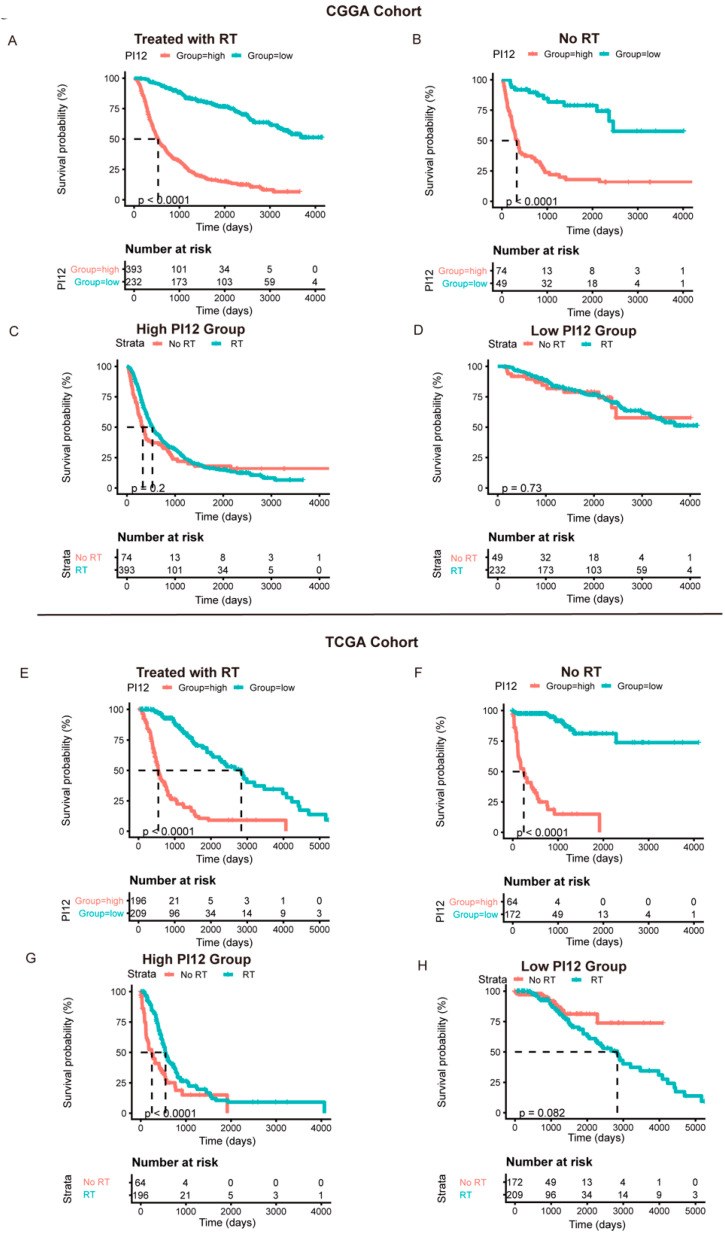
The prognostic value of the risk signature in CGGA cohort and TCGA cohort. (**A**,**E**) Kaplan–Meier survival analysis of the PI12 score for glioma patients treated with radiotherapy in CGGA cohort and TCGA cohort; (**B**,**F**) Kaplan–Meier survival analysis of the PI12 score for glioma patients without radiotherapy in CGGA cohort and TCGA cohort; (**C**,**G**) Kaplan–Meier survival analysis of radiotherapy for glioma patients in high-PI12 group in CGGA cohort and TCGA cohort; (**D**,**H**) Kaplan–Meier survival analysis of radiotherapy for glioma patients in low-PI12 group in CGGA cohort and TCGA cohort.

In the CGGA cohort, we tested the specificity of the PI12 using the Cox analysis of the RT and non-RT groups. Promising radiosensitivity predictive indexes were expected to distinguish between outcomes in the RT group, without influencing the outcome of the no-RT group. However, PI12 revealed prognostic potential on outcome in the RT and non-RT groups (Figure 6A,B). As for the sensitivity of PI12 score, no significant differences were seen between the RT and non-RT groups for patients in low- and high-PI12 groups (Figure 6C,D).

In the TCGA cohort, PI12 also displayed an obviously prognostic value whether or not participants received radiotherapy (Figure 6E,F). However, for the high-PI12 group, patients with RT tended to have better outcomes than those without RT, while for the low-PI12 group, no significant differences were seen between the patients with and without RT.

**Figure 7:**
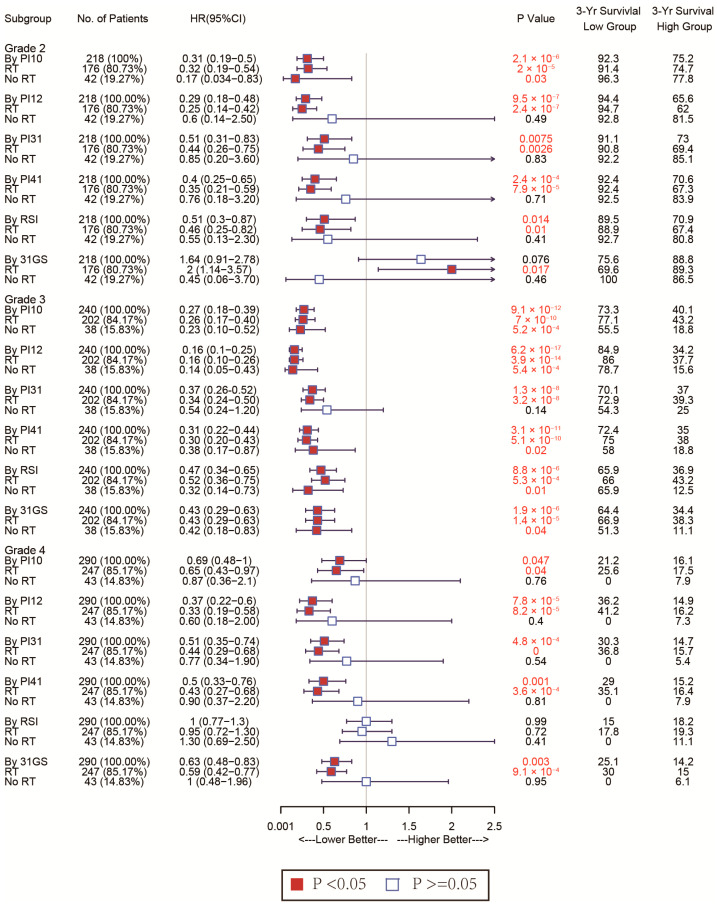
Forest plots to test the specificity of the selected markers for different WHO stages in the CGGA cohort.

**Figure 8:**
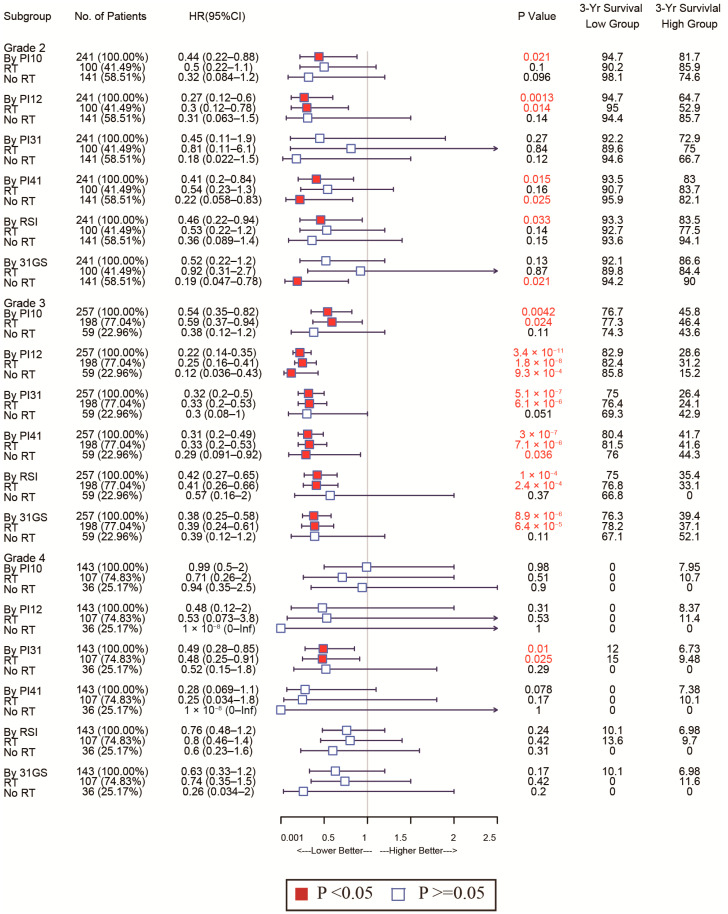
Forest plots to test the specificity of the selected markers for different WHO stages in the TGGA cohort.

Given glioma’s heterogeneity, we stratified the patients based on WHO classification and tested discrimination capability in various glioma grades. The forest plot in Figure 7 shows that of the CGGA cohort. For WHO grades 2 and 4, patients with low PI12 levels exhibited better survival after RT, while no significant differences were seen between the low PI31 group and high PI31 group in patients who not receive RT. In the WHO grade 3 group, PI12 showed an obviously prognostic value whether or not participants received RT. In the TCGA cohort, PI12 showed its discrimination capability only for the patients with WHO grade 2, but it failed for those with WHO grade 3 and 4 (Figure 8).

In all WHO tumor grades of the CGGA cohort, patients with low PI31 levels exhibited better survival after RT. No significant differences were seen between the low PI31 group and high PI31 group in patients who did not receive RT. PI31 also showed its discrimination capability in those with WHO grades 3 and 4 in the TCGA cohort. Other radiosensitivity predictive indexes did not exhibit specificity like PI31 and PI12, which are shown in Figure 7 and Figure 8.

**Figure 9:**
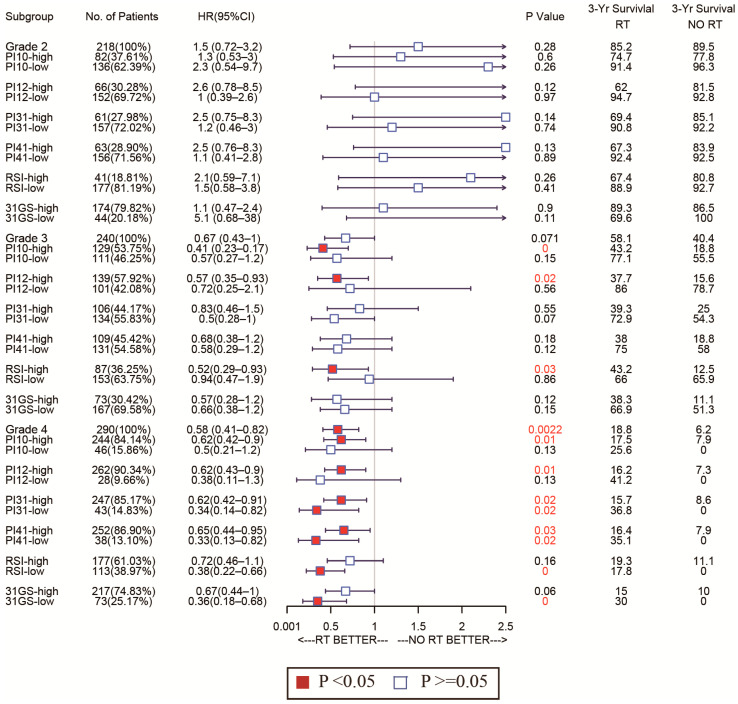
Forest plots to test the sensitivity of the selected markers for different WHO stages in the CGGA cohort.

**Figure 10:**
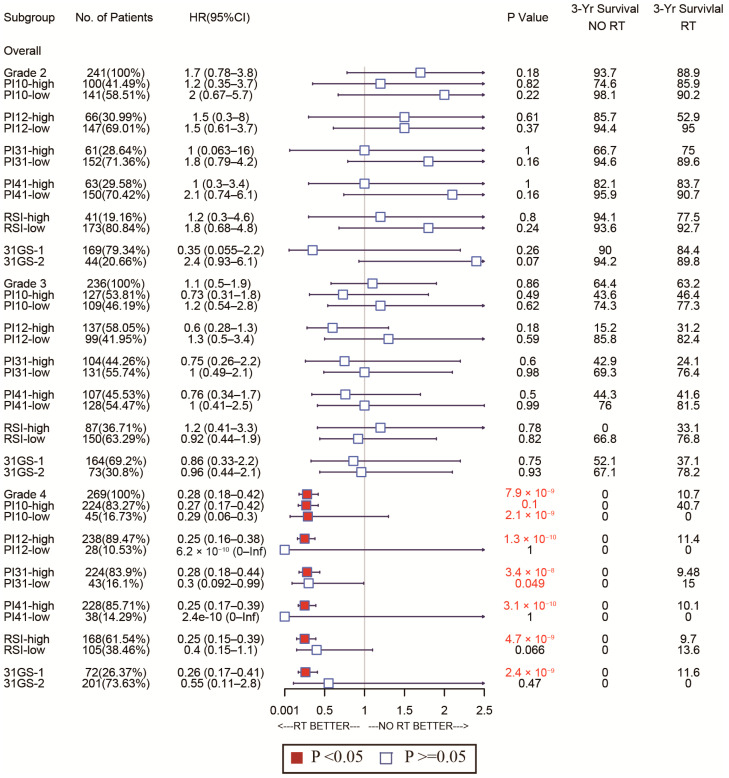
Forest plots to test the sensitivity of the selected markers for different WHO stages in the TCGA cohort.

As for the sensitivity of the selected markers, we also used forest plots to display this for different subtypes. In the CGGA cohort, the high-PI12 group could benefit from RT, while the low-PI12 group could not for the patients with WHO grade 3 and 4 (Figure 9). In the TCGA cohort, PI12 detected that high-PI12 group patients could benefit from RT only for those with WHO grade 4 (Figure 10).

### 3.5. Construction of a Nomogram for Predicting Glioma OS

**Figure 11:**
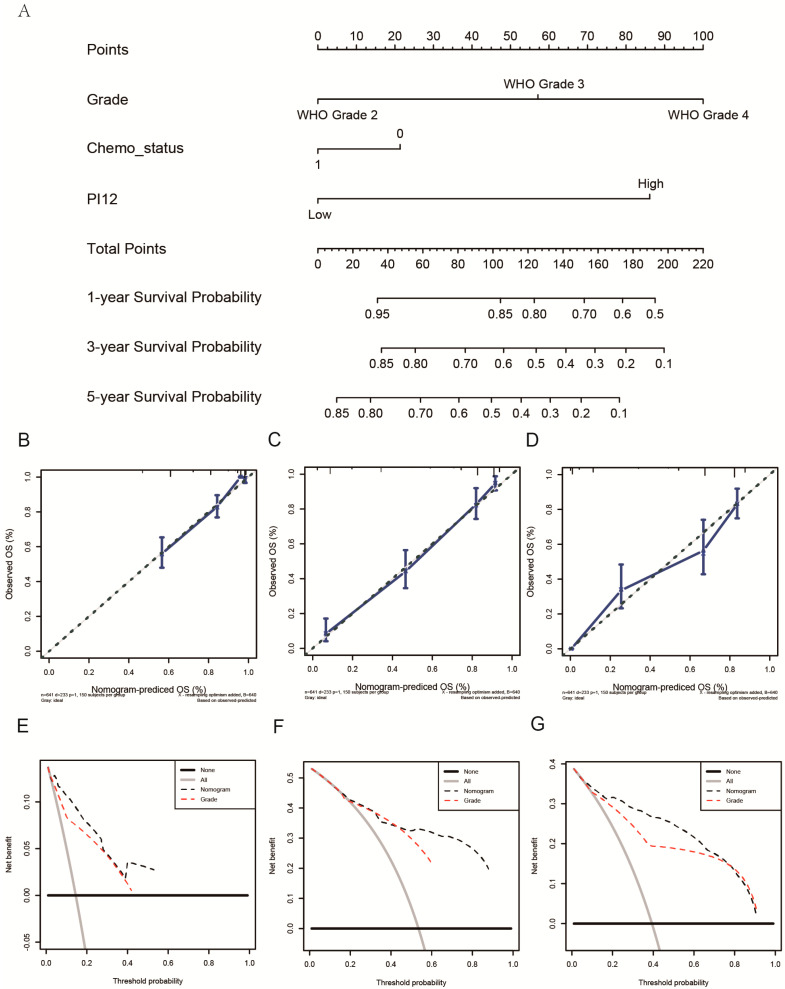
Construction and validation of a nomogram. (**A**) The nomogram predicting 1−, 3− and 5−year survival. (**B**–**D**) Calibration plots of the nomogram showed that the predicted 1−, 3− and 5−year survival probabilities of the validated cohort for OS were almost identical to the actual observations. (**E**–**G**) Decision curve analysis from the validation set for 1-, 3- and 5-year survival.

As PI12 exhibited significant superiority in survival prediction in both cohorts, we used it as a variable in nomogram construction. After univariate and multivariate analysis of the CGGA dataset (training cohort), only PI12, WHO grade, and chemotherapy remained (Table 3). A nomogram was then constructed based on the selected variables to predict the 1-, 3-, and 5-year survival rates (Figure 11A). We then validated the nomogram externally through C-index, calibration plots, and decision curve analyses of the validation cohort. The nomogram exhibited a significantly higher OS C-index relative to the WHO’s staging system (0.842 vs. 0.787, *p* ≤ 2.2 × 10^−16^). Nomogram calibration plots showed that the predicted 1-, 3- and 5-year survival probabilities in the validated cohort were almost identical to actual observations (Figure 11B–D). Decision curve analysis showed that the net benefits indicated by the nomogram were higher than those from the WHO’s staging system (Figure 11E–G).

### 3.6. Exploration of Biological Function

In order to explore the functional characteristics of potential changes associated with radiosensitivity, GO analysis was conducted to investigate the differences in biological processes between low- and high-PI12 groups. We identified 276 up-regulated genes and 44 down-regulated genes (Figure 1A–D). Genes up-regulated in the high-PI12 group were primarily involved in cell division and proliferation, including “Mitotic nuclear division”, “Nuclear division”, “Mitotic sister chromatid segregation”, “Organelle fission” and “Regulation of mitotic nuclear division” (Figure 1E). The KEGG pathways that were particularly enriched by up-regulated DEGs were “Cell cycle”, “Cellular senescence”, “PPAR signaling pathway” and “P53 signaling pathway” (Figure 1F). Genes that were negatively correlated with high PI12 were enriched in biological processes associated with regulation of membrane potential and transmembrane transporter activity (Figure 1G). Down-regulated genes were also enriched in KEGG pathways such as “insulin secretion”, “GABAergic synapse”, “neuroactive ligand receptor interaction” (Figure 1H). GSEA analyses were carried out for validation. The results suggests that the high-risk groups were related to the regulation of glycolysis metabolism and hypoxic response. The interferon-γ response also had a significant effect (Figure 1I–N).

**Figure 12:**
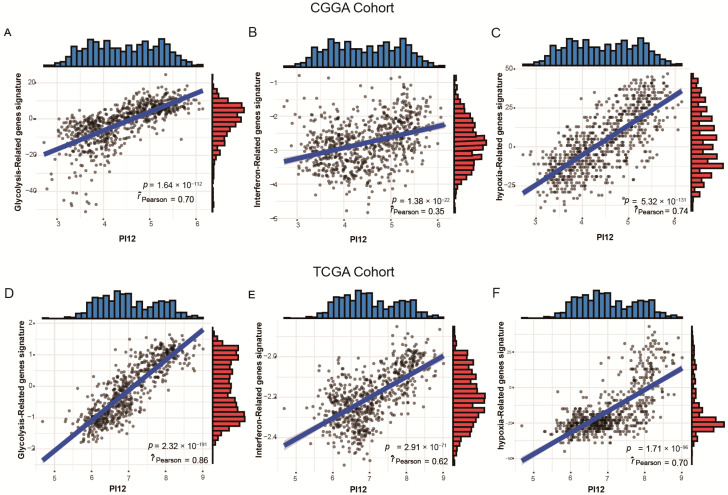
Relationship between PI12 score and validated biological process signatures. (**A**,**D**) Correlation between PI12 score and glycolysis−related genes signatures; (**B**,**E**) Correlation between PI12 score and interferon-related gene signatures; (**C**,**F**) Correlation between PI12 score and interferon-related gene signatures.

To validate the association between PI12 and related biological processes, we investigated the relationship between the PI12 score and published gene signatures. Overall, our PI12 score was strongly correlated to the glycolysis-related gene signatures, shown in Figure 12A,D (r = 0.70, *p* < 0.05 for the CGGA cohort; r = 0.86, *p* < 0.05 for the TCGA cohort); the PI12 score was also strongly correlated to the hypoxia score, shown in Figure 12B,E (r = 0.74, *p* < 0.05 for the CGGA cohort; r = 0.70, *p* < 0.05 for the TCGA cohort). However, the PI12 score was weakly correlated to interferon signature, as shown in Figure 12C,F (r = 0.35, *p* < 0.05 for the CGGA cohort; r = 0.62, *p* < 0.05 for the TCGA cohort).

## 4. Discussion

Glioma, the most common brain malignancy, is characterized by a high relapse and mortality rate [16]. Due to its biological heterogeneity, glioma exhibits great variability in survival, ranging from weeks/months to years [2]. Conventional glioma grading is based on histological morphology. However, the accuracy of this approach is greatly affected by interobserver variability and insufficient tissue sampling [17]. Recent studies have uncovered key genetic and molecular aspects of glioma, leading to the identification of novel prognostic biomarkers that are superior to traditional histological methods of diagnosing, staging, and treating glioma. The IDH mutation status is a hallmark of glioma prognosis signatures. IDH wild-type LGGs are generally as aggressive as GBM and have poor prognosis. IDH wild-type GBM also has a poorer prognosis relative to IDH mutant cases. Chromosome 1p/19q co-deletion is a well-known prognostic and predictive marker of grade 2 and 3 glioma [18]. MGMT (O6-methylguanine-DNA methyltransferase) promoter methylation is also an important prognostic, and predictive factor in GBM. MGMT elevation may cause temozolomide resistance [19]. Other potential GBM biomarkers include PD-1 (programmed death 1), which is also a therapeutic target of immune checkpoint inhibitors [20]. Although radiotherapy is the most commonly used and cost-effective adjunct therapy for glioma, there are no effective predictors to guide radiotherapy.

The radiosensitivity index (RSI) is a 10-gene model for predicting tumor radiosensitivity. This model uses a linear regression algorithm that correlates gene expression and intrinsic cellular radiosensitivity, represented by the SF2 in 48 human cancer cell lines [4]. The RSI has been shown to predict radiosensitivity in various tumors [4,5,6,7]. An advantage of RSI is the quantifiability of the linear regression algorithm, making it more intuitive than unsupervised methods. A genomic-adjusted radiation dose (GARD) model based on RSI is used to tailor radiotherapy doses to meet individual needs [21] in breast cancer and lung metastases of various primary tumors [22,23]. However, in GBMs, RSI is an independent prognostic factor for patients, irrespective of RT status [24]. To the best of our knowledge, RSI has not been tested in LGG. Here, we find that RSI is a significant prognostic factor in glioma cases. However, subgroup analysis had inconsistent results in the CGGA and TCGA cohorts and showed unfavorable sensitivity and specificity when RSI was used to predict radiosensitivity in WHO grade 2−4 gliomas.

The 31-GS was developed from the SF2 of the NCI-60 cancer cell panel. Four published NCI-60 cancer cells microarray datasets were used, and meta-analysis was used to integrate a new radiosensitivity signature with 31 genes [8]. The 31-GS has been validated for GBM radiosensitivity prediction [25]. However, for LGGs, breast cancers, and head and neck cancers, PD-1 expression should be combined with the 31-GS to predict radiosensitivity [9,10,11]. The 31-GS uses unsupervised learning to stratify patients into RR and RS groups. However, discriminating gene signatures using unsupervised methods is difficult. Thus, more effective and accurate classifiers are needed for clinical use with 31-GS. Additionally, because it is unquantifiable, the 31-GS cannot predict individual doses such as RSI. Here, we find that the 31-GS is an independent prognostic factor only in the RT group and could not predict the survival difference between RS and RR groups in patients without RT, thus failing to identify patients who may benefit from RT.

Both RSI and 31-GS have limitations that curtail their potential to predict radiosensitivity and inform clinical decisions. Moreover, since they are based on numerous cancer types, adapting them to glioma may improve their capacity to predict radiosensitivity. Thus, we built four linear regression algorithms using genes from 31-GS, RSI, or both, based on the training cohort. Although the 31-GS and RSI are non-overlapping, some genes strongly correlated with each other. LASSO regression was used to shrink the number of key genes, producing 12 genes that were included in the development of a new biomarker—PI12.

Here, we have presented the first comparison of the ability of 31-GS and RSI to predict glioma prognosis. AUC, NRI, and IDI values of the 31-GS were higher than those of RSI at all time points. Comparison of all newly established predictors showed that PI12 had the highest AUC, NRI, and IDI in the training and validation cohorts at most time points, indicating good prognostic potential.

Forest plot analysis of these biomarkers’ power to predict radiosensitivity showed that all have prognostic potential in the training and validation cohorts, irrespective of RT status. In the training cohort, none of the biomarkers could identify the subgroup whose patients could benefit from RT. However, in the validated cohort, PI31, 31-GS, and RSI identified the high-risk group that could benefit from RT and the low-risk group that could benefit from being exempted from RT. The PI12, PI10, and PI41 could only identify the subgroup that may benefit from RT. The divergences observed in the two cohorts failed to support the clinical use of these biomarkers. However, due to glioma’s heterogeneity, therapeutic strategies vary by disease grade. Subgroup analysis of the radiosensitivity discriminating power of these biomarkers in WHO grade 2–4 gliomas showed that, relative to other biomarkers, PI12 and PI31 had superior sensitivity and specificity in WHO grades. Our data show that PI12 is more suited for WHO glioma grade 2, while PI31 is better for grades 3 and 4. While 31-GS and RSI are established biomarkers, they are pan-cancer-based. Our data indicate that the adapted gene signatures improve our capacity to predict glioma radiosensitivity.

Further, the aim of a radiosensitivity predictor is to identify patients with unmatched ideal and actual treatment strategies, including the following types: (1) patients with high radiosensitivity that can be considered to reduce the therapeutic dose; (2) patients that have the least aggressive tumors and remain recurrence-free even without RT; (3) patients with radio-resistance that require seeking other adjuvant therapies or increasing local dose; (4) patients with radio-resistance that do not benefit from radiotherapy. The goal of this study was to construct a predictive model to distinguish between the above scenarios. Subgroup analysis revealed that WHO grade 2 patients did not benefit from radiotherapy regardless of whether they were in the high- or low-PI12 group, possibly due to a failure to adequately consider patient age, extent of tumor resection, IDH, and 1p/19q status, but the limited sample size did not support a more detailed subgroup analysis. PI12 failed to identify patients who could be exempted from radiotherapy or reduced dose but targeting the high PI12 group may mean a worse prognosis and require more attention, such as more frequent follow-up observations. WHO grade 3 and 4 patients should be treated with radiation regardless of complete tumor resection, and patients in these grades who have a high level of PI12 may benefit from radiation therapy, while low-PI12 patients do not. However, due to the high recurrence rate and poor prognosis of aggressive gliomas, it cannot be confidently assumed that low-PI12 patients can be exempted from radiotherapy, and it may be more clinically relevant to find other adjuvant therapies, such as checkpoint blockade immunotherapy and electric field therapy, or to find therapeutic targets that enhance radiosensitivity.

Our findings show that WHO glioma grade, chemotherapy status, and PI12 are independent glioma prognostic factors. To complement traditional classification, we established a nomogram based on these factors and evaluated its predictive accuracy and discriminative capacity using C-index (concordance index) analysis. The nomogram’s higher C-index relative to the WHO grading system suggested that it had superior prognostic potential. Furthermore, decision curve analysis plots revealed the benefits of the nomogram.

In the present study, bioinformatics analysis suggested that risk score was positively related to several biological processes such as glycolysis, hypoxia response and interferon gamma response. Glycolysis rate is obviously higher in tumor cells compared to normal cells.

Hypoxia is present to some extent in most solid malignant human cancers [26], and there is sufficient evidence that tumor cells adapted to hypoxic conditions are a major factor in tumor radioresistance [27,28]. Buffa et al. established a hypoxic score to predict outcome and benefit from particular interventions [13]. We proved that our radiosensitivity predictive index-PI12 was obviously correlated to the Buffa hypoxic score. Meanwhile, hypoxia is considered to be an important factor leading to tumor metabolic reprogramming [29]. Increased glycolysis plays an essential role in the occurrence and development of glioma [30]. Wang et al. established a glycolysis-related signature that was a robust predictor of prognosis. This study also explored the correlation of PI12 and anaerobic fraction and glycolysis-related signature. The results showed that radiosensitivity predictive index was positively correlated with glycolysis-related signature, which was consistent with the results of GSEA analysis [14]. Moreover, the interferon-γ pathway was also up-regulated in high-risk patients and PI12 was moderately positively correlated with interferon-related scores and was considered to be associated with changes in the immune microenvironment [15].

The present study has a few limitations. First, samples were downloaded from the TCGA and CGGA databases, and information on the extent of tumor resection and IDH-1 status were unavailable. As the extent of the tumor resection is a critical survival factor, further analysis with more detailed clinical information is needed. Additionally, our study did not examine the prediction of relapse-free survival (RFS) rate, as RFS details were incomplete. Other limitations include those inherent in retrospective database analyses and differences in survival expectations between people of different races. Further prospective research and multicenter clinical trials are needed to validate and refine the model.

## Figures and Tables

**Table 1:** Characteristics of patients in the CGGA cohort and TCGA cohort.

	CGGA Cohort		TCGA Cohort	
	Number	Percent	Number	Percent
**Sex**				
Female	306	40.9%	276	42.7%
Male	442	59.1%	370	57.2%
Missing	0	0.0%	1	0.2%
**Age**				
<40	292	39.0%	243	37.6%
≥40	456	61.0%	403	62.3%
Missing	0	0.0%	1	0.2%
**WHO classification**				
Grade 2	218	29.1%	244	37.7%
Grade 3	240	32.1%	258	39.9%
Grade 4	290	38.8%	145	22.4%
**Radio status**				
Yes	625	83.6%	406	62.8%
No	123	16.4%	241	37.3%
**Chemo status**				
Yes	520	69.5%	372	57.5%
No	228	30.5%	275	42.5%
**IDH mutant status**				
Mutant	409	54.7%	419	64.8%
Wildtype	339	45.3%	228	35.2%
**X1p19q_codeletion_status**				
Codeletion	155	20.7%	NA	NA
Non-codeletion	593	79.3%	NA	NA

**Table 2:** Radiosensitivity index (41-gene signature) for predicting radiosensitivity.

Genes	Group	Coefficients of Cox Analysis	HR (95% CI for HR)	Wald. Test	*p*-Value	Coefficients of Lasso-Cox Model
CAPNS1	31-GS	0.31	1.4 (1.2–1.5)	33	1.10 × 10^−9^	0.000
PFN2	31-GS	−0.4	0.67 (0.59–0.76)	41	1.30 × 10^−10^	0.000
PYGB	31-GS	−0.56	0.57 (0.48–0.68)	41	1.40 × 10^−10^	−0.173
SCRN1	31-GS	−0.078	0.92 (0.82–1)	1.6	0.21	0.000
CCND1	31-GS	0.051	1.1 (0.97–1.1)	1.4	0.23	0.022
HTRA1	31-GS	−0.38	0.69 (0.6–0.79)	28	1.00 × 10^−7^	−0.170
ITGB5	31-GS	0.33	1.4 (1.2–1.5)	37	1.30 × 10^−9^	0.000
RALB	31-GS	0.39	1.5 (1.3–1.7)	23	1.50 × 10^−6^	0.000
TWF1	31-GS	0.27	1.3 (1.1–1.5)	13	0.00027	0.069
ANXA2	31-GS	0.36	1.4 (1.4–1.5)	170	8.80 × 10^−39^	0.000
PKM	31-GS	0.53	1.7 (1.4–2)	38	8.60 × 10^−10^	0.000
ACTN1	31-GS	0.41	1.5 (1.4–1.6)	140	2.10 × 10^−32^	0.105
ANXA5	31-GS	0.69	2 (1.8–2.2)	160	2.00 × 10^−36^	0.103
PTMS	31-GS	0.27	1.3 (1.2–1.5)	24	8. × 10^−7^	0.084
CD63	31-GS	0.53	1.7 (1.5–1.9)	120	1.90 × 10^−27^	0.288
RAB13	31-GS	0.61	1.8 (1.6–2.1)	100	4.80 × 10^−24^	0.000
CBR1	31-GS	0.28	1.3 (1.2–1.4)	46	1.20 × 10^−11^	0.071
SQSTM1	31-GS	0.6	1.8 (1.6–2.1)	71	2.90 × 10^−17^	0.000
HCLS1	31-GS	0.39	1.5 (1.3–1.6)	70	5.80 × 10^−17^	0.000
PTPRCAP	31-GS	0.0041	1 (0.92–1.1)	0.01	0.92	−0.104
PTPRC	31-GS	0.21	1.2 (1.1–1.3)	24	1.10 × 10^−6^	0.000
CORO1A	31-GS	0.25	1.3 (1.1–1.4)	20	9.10 × 10^−6^	0.000
ARHGDIB	31-GS	0.52	1.7 (1.5–1.8)	100	1.60 × 10^−24^	0.000
LAPTM5	31-GS	0.31	1.4 (1.2–1.5)	48	3.60 × 10^−12^	0.000
WAS	31-GS	0.29	1.3 (1.2–1.5)	31	2.30 × 10^−8^	−0.014
CXCR4	31-GS	0.34	1.4 (1.3–1.5)	74	6.20 × 10^−18^	0.000
LRMP	31-GS	0.07	1.1 (0.92–1.3)	0.8	0.37	0.000
DAG1	31-GS	0.3	1.4 (1.2–1.6)	14	2.2 × 10^−4^	0.000
PIR	31-GS	0.17	1.2 (1.1–1.3)	12	5.1 × 10^−4^	0.000
MYB	31-GS	0.36	1.4 (1.3–1.6)	28	1.40 × 10^−7^	0.000
EMP2	31-GS	0.36	1.4 (1.3–1.6)	49	2.60 × 10^−12^	0.000
AR	RSI	0.36	1.4 (1.3–1.6)	30	3.50 × 10^−8^	0.000
JUN	RSI	0.51	1.7 (1.5–1.8)	120	5.20 × 10^−28^	0.000
STAT1	RSI	0.32	1.4 (1.2–1.5)	43	6.50 × 10^−11^	0.000
PRKCB	RSI	−0.29	0.75 (0.69–0.81)	51	8.10 × 10^−13^	0.000
RELA	RSI	0.35	1.4 (1.1–1.8)	9.9	0.0017	0.000
ABL1	RSI	0.19	1.2 (1–1.4)	6.2	0.013	0.000
SUMO1	RSI	0.8	2.2 (1.7–2.9)	39	4.60 × 10^−10^	0.000
PAK2	RSI	0.042	1 (0.89–1.2)	0.27	0.6	0.000
HDAC1	RSI	0.86	2.4 (2–2.7)	130	3.90 × 10^−31^	0.331
IRF1	RSI	0.37	1.4 (1.3–1.6)	68	2.10 × 10^−16^	0.000

**Table 3:** Univariate analysis and multivariate analysis of the training cohort.

	Univariate Analysis			Multivariate Analysis			
	HR	95%CI	*p*-Value	HR	95%CI	*p*-Value	
**Sex**							
Female	Reference						
Male	0.038	0.86–1.3	0.35				
**Age**							
<40	Reference			Reference			
≥40	1.6	1.3–2.0	1.8 × 10^−6^	1.18	0.96–1.44	0.11	
**WHO classification**							
Grade 2	Reference			Reference			
Grade 3	2.1	2.2–3.9	<3.5 × 10^−54^	2.56	1.87–3.49	3.45 × 10^−14^	
Grade 4	2.1	6.3–11	<3.5 × 10^−54^	4.66			
**Radio status**							
Yes	Reference						
No	0.93	0.72–1.2	0.57				
**Chemo status**							
Yes	Reference						
No	1.6	1.3–2.0	5.7 × 10^−6^	0.70	0.56–0.89	3 × 10^−3^	
**IDH mutant status**							
Mutant	Reference			Reference			
Wildtype	3.2	2.6–3.8	3.8 × 10^−32^	1.16	0.93–1.46	0.18	
**X1p19q_codeletion_status**							
Codeletion	Reference			Reference			
Non-codeletion	4.3	3.2–5.9	2.1 × 10^−20^	1.29	1.88–1.91	0.18	
**PI12**							
High	Reference			Reference			
Low	0.16	0.12–0.2	1.5 × 10^−48^	0.28	0.20–0.39	3.05 × 10^−14^	

## Data Availability

The datasets generated and/or analyzed during the current study are available in the UCSC Xena (https://xenabrowser.net/datapages/?cohort=TCGA%20lower%20grade%20glioma%20and%20glioblastoma%20(GBMLGG)&removeHub=https%3A%2F%2Fxena.treehouse.gi.ucsc.edu%3A443) and the Chinese Glioma Genome Atlas (http://www.cgga.org.cn/download.jsp, BIGD accession number: PRJCA001746 and PRJCA001747, accessed on 9 June 2019).
